# Getting to the point—the use of radiofrequency and pulsed field ablation for ablating a challenging redo accessory pathway using an all-in-one dual energy wide area lattice-tip catheter: a case report

**DOI:** 10.1093/ehjcr/ytag279

**Published:** 2026-04-21

**Authors:** Richard Balasubramaniam, Fazlullah Wardak, Wessam Ali, Candice Henry, Mark Sopher

**Affiliations:** Royal Bournemouth Hospital, University Hospitals Dorset, Castle Lane East, Bournemouth BH7 7DW, United Kingdom; Royal Bournemouth Hospital, University Hospitals Dorset, Castle Lane East, Bournemouth BH7 7DW, United Kingdom; Royal Bournemouth Hospital, University Hospitals Dorset, Castle Lane East, Bournemouth BH7 7DW, United Kingdom; Royal Bournemouth Hospital, University Hospitals Dorset, Castle Lane East, Bournemouth BH7 7DW, United Kingdom; Royal Bournemouth Hospital, University Hospitals Dorset, Castle Lane East, Bournemouth BH7 7DW, United Kingdom

## Case description

A 49-year-old man with palpitations presented to the emergency department with supraventricular tachycardia (SVT). The patient had undergone 3 previous radiofrequency (RF) ablations to eliminate a concealed left lateral accessory pathway, but tachycardia recurred within 2 weeks each time despite apparent rapid procedural ablation success.

A further procedure was performed with a dual energy wide area lattice-tip catheter (Sphere-9) and 3D mapping (Affera). Electrophysiology study revealed eccentric, non-decremental retrograde conduction during ventricular pacing suggestive of pathway recurrence. Antegrade curve was decremental, without pre-excitation, and led to SVT induction with an eccentric retrograde limb, consistent with a left sided pathway-mediated tachycardia. VA time was 80 ms and ventricular entrainment revealed a VAV response with a relatively short delta of the post-pacing interval, all in keeping with SVT via the pathway.^1^

**Figure 1:**
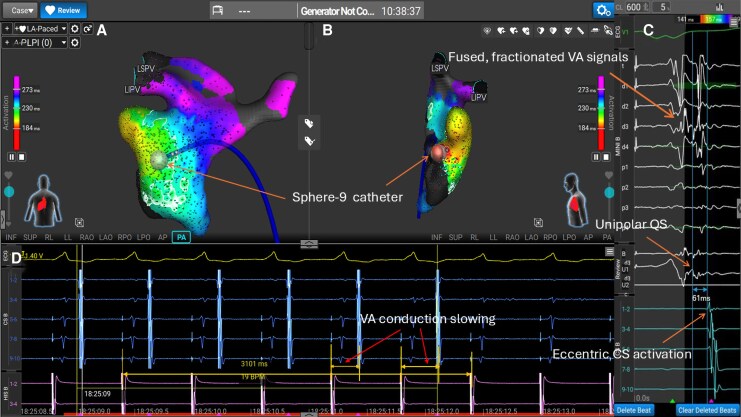
Successful acute radiofrequency (RF) ablation of the left lateral accessory pathway using the sphere-9 catheter and Affera mapping. Positioning of the Sphere-9 catheter at the area of earliest activation on the Affera map is shown in PA (*A*) and left lateral (*B*) projections. Pacing from the right ventricle (*D*, in lilac) reveals pathway conduction with an eccentric VA conduction pattern seen on the CS catheter (in blue, D or expended in cyan (*C*)). A fused and fractionated signal is seen on the Sphere-9 catheter (*C*) with a unipolar QS signal at the point of earliest atrial activation (*C*). With the first application of RF energy from the Sphere-9 catheter, the accessory pathway was successfully ablated with no VA conduction seen after just over 3 s (3101 ms, *D*). Slowing of VA conduction is seen in the paced beat prior to pathway abolishment (*D*). Further consolidation lesions were applied with RF and pulsed field ablation applications delivered on top of the RF applications for a wider and deeper lesion in this redo case.

The Sphere-9 catheter was advanced transeptally to the left atrium and used for high density pathway mapping marking the area of earliest atrial activation with ventricular pacing (*Figure 1*). Identification of discrete areas of early atrial activation was localized to a small segment along the lateral mitral valve annulus where unipolar QS signals were seen on the catheter. RF energy was delivered using standard Affera mitral isthmus settings resulting in prompt VA conduction loss indicating rapid pathway elimination. 4 further consolidation lesions were applied with RF and 3 pulse field ablation applications performed sequentially to increase lesion depth, with nitrates given to avoid coronary artery vasospasm.^2,3^ Post-ablation testing demonstrated no retrograde conduction including with isoproterenol, and non-inducibility of tachycardia with programmed stimulation.

The patient was discharged and has remained arrhythmia-free at 4-month follow-up.

The case highlights the potential value of the Affera mapping system and the Sphere-9 catheter in challenging redo accessory pathway ablations. High-resolution mapping and stable, deep lesion creation with both RF and PF energies can facilitate success in difficult pathway ablation.

## Data Availability

The data underlying this article are available in the article.
